# Invasive Pneumococcal Disease in Adults in Portugal: The Importance of Serotypes 8 and 3 (2015–2018)

**DOI:** 10.3390/microorganisms9051016

**Published:** 2021-05-08

**Authors:** Catarina Silva-Costa, Joana Gomes-Silva, Inês Teodoro, Mário Ramirez, José Melo-Cristino, on behalf of the Portuguese Group for the Study of Streptococcal Infections

**Affiliations:** Faculdade de Medicina, Instituto de Microbiologia, Instituto de Medicina Molecular, Universidade de Lisboa, 1649-028 Lisboa, Portugal; anacosta@fm.ul.pt (C.S.-C.); jfsilva@medicina.ulisboa.pt (J.G.-S.); i.teodoro@campus.ul.pt (I.T.); melo_cristino@fm.ul.pt (J.M.-C.)

**Keywords:** conjugate vaccine, polysaccharide vaccine, serotype, invasive disease, epidemiology, antimicrobial resistance

## Abstract

Increasing the uptake of the 13-valent pneumococcal conjugate vaccine (PCV13) in children is expected to alter the serotypes causing invasive pneumococcal disease (IPD) in adults due to herd protection. We characterized 2172 cases of adult IPD in 2015–2018 in Portugal after the introduction of PCV13 in the national immunization plan of 2015. Among the 58 detected serotypes, serotypes 8 (*n* = 413; 19%), 3 (*n* = 334; 15%), 22F (*n* = 148; 7%), 14 (*n* = 138; 6%), and 19A (*n* = 116; 5%) were the most frequent. Among PCV13 serotypes, 7F and 19A IPD decreased, but serotype 3 IPD remained stable. The non-PCV13 serotypes were a heterogeneous group, with serotypes 23A and 23B enriched among CSF cases; serotype 8 associated with younger patients; and serotypes 22F, 6C, and 31 associated with older patients. The continued increase of serotype 8 IPD was one of the drivers for the increased coverage of the 23-valent pneumococcal polysaccharide vaccine (PPV23; 80% in 2015–2018). Antimicrobial resistance was associated with older age and serotypes 6C, 11A, 14, 15A, 19A, and 19F. Three years after the introduction of PCV13 in the NIP with an uptake of >95%, the proportion of PCV13 serotypes causing IPD in adults stabilized in Portugal. The direct vaccination of adults may be important in preventing IPD in this age group.

## 1. Introduction

The introduction of pneumococcal conjugate vaccines (PCVs) in children led to changes in the incidence and serotypes causing invasive pneumococcal disease (IPD), not only in vaccinated children but also in adults. In fact, initial data from the United States showed that more cases were averted by this herd protection than by the effects on the groups targeted by vaccination. The increasing use of the 23-valent polysaccharide vaccine (PPV23) and the approval of the 13-valent conjugate vaccine (PCV13) for use in adults also has the potential to further impact IPD in this age group. In Portugal, as elsewhere, the use of increasing valency PCVs in children led to changes in the serotypes causing IPD in adults [1,2,3]. However, although reaching a substantial vaccine coverage (around 63%), the use of the PCVs through the private market and without reimbursement may have contributed to the persistence of vaccine serotypes as causes of IPD by creating an uneven distribution of the vaccine. The use of the 7-valent PCV (PCV7) in children led to a decrease in the proportion of PCV7 serotypes in adult IPD in Portugal in 2006–2008, with the most frequent serotypes being 3, 1, 7F, 19A, and 14 [3]. After the introduction of PCV13 for children vaccinations in 2010, a decline in the proportion of the additional serotypes included in PCV13 was observed in adult IPD, mostly driven by decreases in the proportion of serotypes 1, 5, 7F, and 19A, while serotype 3 remained an important serotype in IPD [1,2]. Concomitantly, serotypes not included in any PCV gained importance, with serotype 8 being of special concern and becoming a leading serotype in adult IPD in Portugal [1] and elsewhere [4,5,6]. Other emerging non-PCV13 serotypes in Portugal include the 22F, 11A, 9N, 6C, 15B/C, and 15A serotypes [1].

Despite the reductions in PCV13 serotypes, the fraction of adult IPD that is potentially vaccine-preventable remained high, with data from 2014 showing that PCV13 serotypes significantly persisted (38%) and that isolates expressing PPV23 serotypes accounted for 75% of all adult IPD [1]. Though several medical associations issued age-based recommendations for vaccination with both PCV13 and PPV23 [7], the official guidelines of the Portuguese national health authority recommend vaccination only in risk groups [8]. There is no national data on the uptake of the pneumococcal vaccine in adults in Portugal, but a single-center study conducted in 2007 revealed that <10% of individuals ≥65 years had been vaccinated with PPV23 [9]. In 2015, PCV13 was introduced in the NIP for children, with doses given at 2, 4, and 12 months of age. Despite the expected low uptake of PCV13 in adults, the broader vaccine uptake achieved by universal childhood vaccination could potentially further impact adult IPD serotype distribution. Furthermore, 15- and 20-valent PCVs are applying for approval for use in adults and should reach the market soon [10,11]. The aims of this work were to document serotype changes and susceptibility patterns among pneumococci causing adult IPD following the introduction of PCV13 in the NIP for children and to evaluate the potential benefits of the increased valency vaccines.

## 2. Materials and Methods

Since 1999, the Portuguese Group for the Study of Streptococcal Infections has been monitoring invasive pneumococcal disease in Portugal through a laboratory-based surveillance system of 31 microbiology laboratories involving the collection and shipment of all IPD isolates to a central laboratory for characterization [12]. Though all laboratories were periodically contacted to submit isolates to the central laboratory, no audit was performed to ensure compliance, which may be variable in this type of study. A case of IPD was defined by the isolation of pneumococci from a normally sterile fluid, such as blood, cerebrospinal fluid (CSF), or pleural fluid, or the detection of pneumococcal DNA in a normally sterile body fluid, excluding blood. Isolates included in this study were from adult patients (≥18 years) with IPD between January 2015 and December 2018, with only one isolate from each episode being considered. Isolates were identified as pneumococci by colony morphology on blood agar plates, optochin susceptibility, and bile solubility.

Serotypes were determined by the standard capsular reaction test using the chessboard system and specific sera [13] (Statens Serum Institut, Copenhagen, Denmark). Serotypes were classified into vaccine serotypes, i.e., those included in PCV7 (serotypes 4, 6B, 9V, 14, 18C, 19F, and 23F), PCV13 (all PCV7 serotypes plus 1, 3, 5, 6A, 7F, and 19A, the later also referred to as addPCV13), PCV15 (all PCV13 serotypes plus 22F and 33F, the later also referred to as addPCV15), PCV20 (all PCV15 serotypes plus 8, 10A, 11A, 12F, and 15B, the later also referred to as addPCV20), and PPV23 (all PCV20 serotypes, except serotype 6A, plus serotypes 2, 9N, 17F and 20, the later also referred to as addPPV23), or as non-vaccine serotypes (NVTs). Given the high frequency of spontaneous switching between serotypes 15B and 15C, we decided to group isolates with these serotypes into a single group and to assume that PCV20 and PPV23 protect against both these serotypes [14]. Additionally, given the difficulties in phenotypically distinguishing isolates of serotypes 25A and 38, as well as isolates with serotypes 29 and 35B, these also grouped together into 25A/38 and 29/35B, respectively.

Minimal inhibitory concentrations (MICs) for penicillin and cefotaxime were determined using Etest strips (Biomérieux, Marcy l’Étoile, France). Unless otherwise stated, we used the CLSI-recommended breakpoints for oral penicillin as epidemiological breakpoints that allowed for comparison with previous studies [15]. Isolates were further characterized by determining their susceptibility to erythromycin, clindamycin, vancomycin, linezolid, tetracycline, levofloxacin, trimethoprim–sulfamethoxazole, and chloramphenicol by the Kirby–Bauer disk diffusion technique according to the CLSI recommendations and interpretative criteria [15].

Macrolide resistance phenotypes were identified using a double disc test with erythromycin and clindamycin, as previously described [16]. Simultaneous resistance to erythromycin and clindamycin defines the MLS_B_ phenotype (resistance to macrolides, lincosamides, and streptogramin B), while non-susceptibility to only erythromycin indicates the M phenotype.

Simpson’s index of diversity (SID) and respective 95% confidence intervals (CI95) were used to measure population diversity [17]. Differences were evaluated by Fisher’s exact test, and the Cochran–Armitage test (CA) was used for trends with the false discovery rate (FDR) correction for multiple testing [18]. A *p*-value of < 0.05 was considered significant for all tests.

## 3. Results

### 3.1. Isolate Collection

Between 2015 and 2018, a total of 2172 isolates responsible for adult IPD were collected in Portugal, distributed as follows: 529 in 2015, 501 in 2016, 576 in 2017, and 566 in 2018. One additional case was identified and directly serotyped by molecular methods from patient CSF in 2015. In most cases, the pneumococcus was identified in blood (93.1%; *n* = 2013), as well as in the CSF (5.2%; *n* = 113), pleural fluid (1.3%; *n* = 29), peritoneal fluid (0.5%; *n* = 11), synovial fluid (0.2%; *n* = 5), and pericardial fluid (0.1%; *n* = 2). The distribution of the cases among the different age groups was as follows: 397 isolates were recovered from patients aged 18–49 years, 497 were recovered from patients 50–64 years, and 1279 were recovered from older patients (≥65 years).

### 3.2. Serotype Distribution

**Figure 1:**
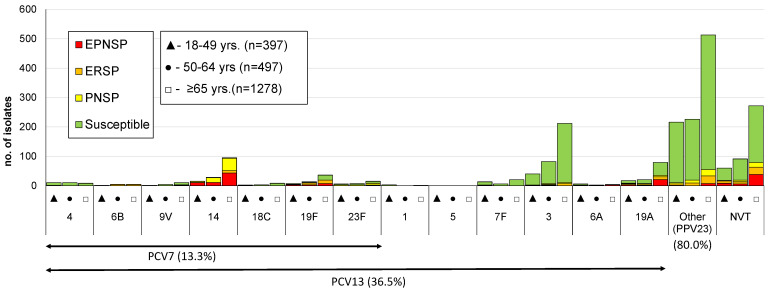
Serotypes of isolates causing invasive pneumococcal disease in adult patients (≥18 years) in Portugal from 2015 to 2018. The number of isolates expressing each serotype in each of the considered age groups is indicated. Isolates recovered from patients 18–49, 50–64, and ≥65 years old are indicated by black triangles, black circles, and open squares, respectively. Isolates presenting both erythromycin resistance and penicillin non-susceptibility (EPNSP) are represented by red bars. Erythromycin-resistant pneumococci (ERP) is indicated by orange bars. Penicillin non-susceptible isolates (PNSP) are indicated by yellow bars. Isolates susceptible to both penicillin and erythromycin are represented by green bars. In one case in the ≥65 years age group, the antimicrobial susceptibility was unknown because the diagnosis was made by molecular methods and is not represented in Figure 1. The serotypes included in the 7-valent conjugate vaccine (PCV7) and the 13-valent conjugate vaccine (PCV13) are indicated by the arrows. NVT: non-vaccine serotypes; other: the additional serotypes included in the 23-valent polysaccharide vaccine (PPV23) and not present in PCV13.

**Figure 2:**
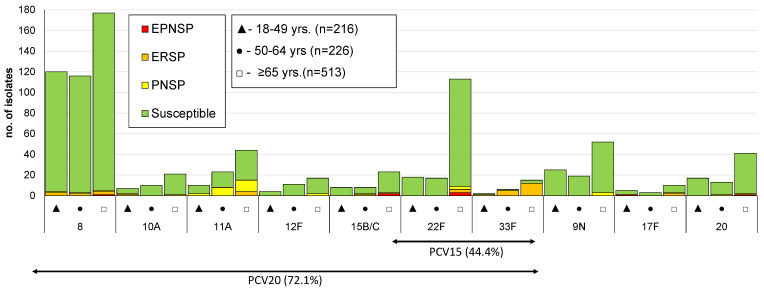
Isolates expressing serotypes present in PPV23 but not included in PCV13 causing invasive pneumococcal disease in adult patients (≥18 years) in Portugal from 2015 to 2018. See legend of Figure 1. Out of the 11 serotypes present in PPV23 but absent from PCV13, serotype 2 was not found in our collection.

**Figure 3:**
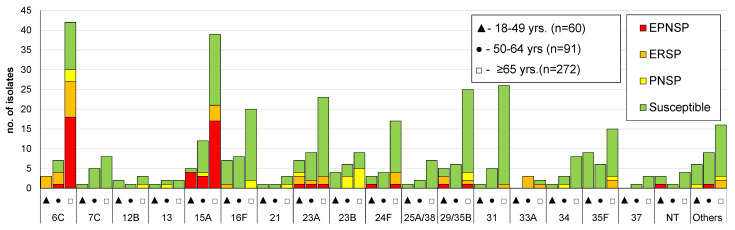
Isolates expressing serotypes not included in any pneumococcal vaccine causing invasive pneumococcal disease in adult patients (≥18 years) in Portugal from 2015 to 2018. See legend of Figure 1. NT: non-typable. Isolates expressing serotypes 25A/38 and 29/35B could not be phenotypically distinguished and are represented together. Only serotypes including *n* > 3 isolates were discriminated. Others include the following serotypes: 9A, 11D (*n* = 3 each); 6D, 11F, 11C, 12A, 15F, 18A, 22A, and 35A (*n* = 2 each); and 7B, 19B, 19C, 24A, 24B, 28A, 39, and 43 (*n* = 1 each).

Overall, 58 different serotypes, as well as eight non-typeable (NT) isolates, were identified. The case identified directly from patient CSF could not be resolved to the serotype level having been identified as 22F/22A and being considered an NVT for the purpose of further analysis. The most frequent serotypes were, by decreasing order, serotypes 8 (*n* = 413; 19%), 3 (*n* = 334; 15%), 22F (*n* = 148; 7%), 14 (*n* = 138; 6%), and 19A (*n* = 116; 5%), together accounting for over half of the isolates (*n* = 1149; 53%). A significant proportion of the isolates still expressed serotypes included in PCV7 (13%; *n* = 289), while over a third of cases were caused by serotypes included in PCV13 (36%; *n* = 794). Serotypes exclusively found in PPV23 were responsible for 44% of IPD cases (*n* = 955) and 20% of the isolates (*n* = 424) expressed serotypes not included in any vaccine formulation (NVTs). The numbers of isolates expressing serotypes included in PCVs and in PPV23, stratified by age group, are represented in Figure 1, Figure 2 and Figure 3. A total of 35 serotypes were found among the 113 CSF isolates, with serotypes 3 (*n* = 22); 19F (*n* = 8); 8 (*n* = 8); 23A (*n* = 7); 22F (*n* = 6); and 6C, 11A, and 23B (*n* = 5 each) represented by ≥5 isolates and together accounting for 58% of all CSF isolates. Out of these, serotypes 19F (*p* = 0.007), 23A (*p* = 0.003), and 23B (*p* = 0.002) were associated with isolation from the CSF (all significant after FDR), while serotype 8 (*p* = 0.001, which was significant after FDR) was underrepresented among CSF isolates.

Overall serotype diversity was high (0.920, CI95 = 0.913–0.926), with no differences in SID between the four years included in this study. Serotype diversity was also high among the three age groups, although a difference was noted between isolates recovered in patients aged 18–49 years (SID = 0.884; CI95 = 0.859–0.908) and those aged ≥65 years (SID = 0.927; CI95 = 0.920–0.933) (*p* = 0.015). Isolates recovered from patients aged 50–64 years had an intermediate SID value between these two: 0.905 (CI95 = 0.888–0.921).

There were differences in serotype distribution between the age groups. The proportion of isolates expressing the 24 major serotypes (*n* > 20 isolates) in each age group is represented in Table 1. Among these serotypes, some decreased in importance with increasing age group, such as serotype 8 and 4, while the opposite trend was noted for serotypes 3, 22F, 6C, 14, and 31 (all significant after FDR correction).

**Figure 4:**
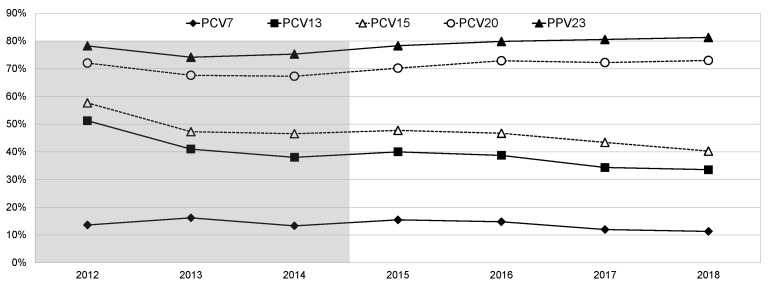
Proportion of isolates expressing serotypes included in existing and future pneumococcal vaccines causing invasive pneumococcal disease in adult patients (≥18 years) in Portugal from 2012 to 2018. The data up to 2014 were presented previously [1]. See text for the serotypes included in each of the vaccines.

During the study period, there were also changes in the proportion of IPD cases caused by vaccine serotypes, as represented in Figure 4. PCV13 serotype IPD decreased from 40.0% in 2015 to 33.6% in 2018, mostly driven by a decrease in the proportion of PCV7 serotype IPD, which decreased from 15.5% in 2015 to 11.3% in 2018, both of which were significant after FDR correction (*p* = 0.01 and *p* = 0.02, respectively). The proportion of IPD caused by isolates expressing addPCV13 serotypes also decreased, though less significantly, from 24.5% in 2015 to 22.3% in 2018. The proportion of PPV23 serotype IPD slightly increased from 78.3% in 2015 to 81.3% in 2018, with the cases caused by the addPCV15, addPCV20, and addPPV23 serotypes considered together increasing from 39.1% in 2015 to 48.4% in 2018 (*p* < 0.001, which was significant after FDR correction). The proportion of NVT IPD decreased slightly from 20.9% in 2015 to 18.0% in 2018. The variations in PCV15 serotype IPD closely tracked those of PCV13 serotype IPD, whereas that of PCV20 serotype IPD closely followed that of PPV23 serotype IPD (Figure 4). When also considering the previous study period (2012–2014) in the analysis, the same trends were significant, but, in this case, the decrease in PCV13 serotype IPD was mostly driven by a decrease in the proportion of IPD cases caused by the addPCV13 serotypes from 37.6% in 2012 to 22.3% in 2018 (*p* < 0.0001, which was significant after FDR correction).

The evolution of individual serotypes responsible for IPD in adults from 2012 to 2018 is represented in Table 2 for serotypes expressed by >3 isolates in at least one of the considered years. In the current study period (2015–2018), the proportion of IPD caused by serotypes 8 and 12F increased from 14.9% and 0.9% in 2015 to 22.1% and 2.7% in 2018, respectively, both unsupported after FDR correction. The opposite trend was detected for serotypes 7F (from 3% to 1.2%), 19A (from 6.8% to 2.8%), and 6C (from 3.8% to 1.8%), although none of these were supported after FDR correction. However, when considering data from 2012 to 2018, several significant changes were detected, including an increase in proportion of serotype 8 IPD (from 8.4% to 22.1%; CA *p* < 0.001) and serotype 33F IPD (from 0.2% to 1.6%; CA *p* = 0.001), as well as decreases in the proportion of serotype 7F (from 8.2% to 1.2%; CA *p* < 0.001), 1 (from 3.0% to 0%; CA *p* < 0.001), and 19A IPD (from 9.7% to 2.8%; CA *p* < 0.001).

The distribution of IPD cases over the study period stratified by age group is represented in Table 3. PCV7 serotypes decreased in patients ≥ 50 years, but this was not supported after FDR correction. The addPCV15 serotypes declined in the 18–49 year-old patients but remained stable in older patients. The addPCV20 serotypes increased in all age groups, but this was only supported after FDR correction in the ≥65 years patients. Both the addPPV23 serotypes and the NVTs varied without a consistent trend.

### 3.3. Antimicrobial Susceptibility

Resistance to all antimicrobials tested is summarized in Table 4. Among the collection, 326 isolates (15.0%) were classified as penicillin non-susceptible pneumococci (PNSP), of which 94.8% (*n* = 309) presented low level resistance and 5.2% (*n* = 17) presented high level resistance. Considering current CLSI breakpoints for parenteral penicillin, 19 out of 112 CSF isolates would have been considered resistant (17.0%), and only 0.2% (*n* = 6) non-CSF isolates would have been considered intermediately resistant. Erythromycin resistance was expressed by 322 isolates (14.8%), of which the majority (*n* = 275; 85.4%) presented the MLS_B_ phenotype and 14.6% (*n* = 47) presented the M phenotype. Simultaneous non-susceptibility to penicillin and erythromycin (EPNSP) accounted for 7.9% of the isolates (*n* = 172). Throughout the study period, resistance to erythromycin significantly decreased from 18.5% in 2015 to 10.8% in 2018, and clindamycin resistance decreased from 15.5% in 2015 to 9.9% in 2018 (CA *p* < 0.001 for both). Penicillin, erythromycin, and clindamycin resistance was mostly associated with the ≥65 years group (CA *p* = 0.002, *p* = 0.01, and *p* = 0.007, respectively).

The proportion of resistant isolates among each serotype is illustrated in Figure 1, Figure 2 and Figure 3. The most frequent serotypes (*n* > 20) among penicillin non-susceptible isolates were serotypes 14, 19A, 15A, 6C, and 11A (by decreasing order of frequency), together accounting for 74.2% (*n* = 242) of the isolates. Serotypes 14, 19A, 6C, 19F, and 15A were, by decreasing order of frequency, the most frequent (*n* > 20) among erythromycin-resistant isolates, together accounting for 65.2% (*n* = 210) of the isolates. PCV7 serotypes accounted for 51.3% of PNSP, 27.3% of ERP, and 45.3% of EPNSP (Figure 1), while PCV13 serotypes accounted for 61.0%, 46.7%, and 65.1% for PNSP, ERP, and EPNSP, respectively (Figure 2). Among EPNSP isolates, a considerable proportion expressed NVT (30.2%), and among ERP and PNSP isolates, 26.7% and 15.6%, respectively, expressed NVT. The most frequent NVT among PNSP isolates were 6C, 15A, 23A, and 23B, together accounting for 8% of PNSP, 16% of ERP, and 27% of EPNSP (Figure 3).

## 4. Discussion

It is well established that vaccinating children leads to a decrease of vaccine-type IPD in adults through herd immunity [19,20]. This is thought to be related to the proportion of vaccinated children [19], so the increase in uptake anticipated from the introduction of PCV13 in the NIP in Portugal was expected to result in further effects in adult IPD. Moreover, the emergence of NVT disease could further influence the serotypes in IPD and even partially erode the benefits of the herd effect of vaccination on the overall burden of disease [20,21].

The decrease of PCV7 serotypes, felt since a few years after PCV7 introduction [1,2], continued to occur but at a very slow pace and with all PCV7 serotypes still being present as causes of IPD in 2018. In contrast, the decrease of the addPCV13 serotypes was underpinned by serotypes 1 and 5, which were not found among IPD cases in the last years of the study, and decreases in serotypes 7F and 19A. In fact, the decrease in addPCV13 serotypes was not more pronounced due to an increase in serotype 3 IPD. Significant increases in the incidence of serotype 3 IPD were also seen in France and England in recent years [21,22]. In fact, the leading serotypes causing adult IPD in Portugal in 2015–2018 were similar to those found in other European countries [4,21,22,23], although their rank order could be different. Among the PCV serotypes, serotype 3 was always found to be a leading cause of adult IPD in Europe in recent years, together with serotypes 19A and 7F [4,21,22,23], although the latter two have frequently been found to be decreasing of late, as seen in Portugal. Serotype 14 persists in Portugal and Spain [4,23] but is less frequent elsewhere [21,22]. Among the serotypes not included in any PCV, serotype 8 is consistently found among the three leading causes of adult IPD, if not the most prevalent, while serotypes 9N, 12F and 22F are also leading causes of IPD, albeit with a more variable rank order [4,21,22,23]. For instance, serotype 12F is one of the three most prevalent serotypes in Spain and England [4,22,23], but it is much less frequent in Portugal and France [21]. Among the other non-PCV serotypes responsible for >2% of adult IPD in Portugal in 2015–2018—11A, 20, 15A, and 6C—most were also recently found to be important causes of adult IPD in Spain [4,23], and serotype 15A was also important in France and England [21,22], but serotype 20 was not a significant cause of disease in any of these countries. Though we have considered serotype 6C an NVT, cross-protection from the 6A and 6B components in PCV13 has been suggested, leading to a future potential reduction of this serotype despite its current persistence as a cause of adult IPD in several countries. In Ontario, Canada, IPD was found to have a similar serotype distribution to that of these European countries, with a smaller share of serotype 8 IPD [24]. In Argentina, five serotypes are responsible for over 5% of all adult IPD: 3, 8, 12F, 7F, and 1 [25]. Most of these are also among the most frequent in Europe, but, in contrast to Argentina, serotype 1 has greatly decreased or is even absent from adult IPD in European countries. Taken together, these results suggest that a similar set of serotypes is emerging in most countries using PCV13 in children as the leading causes of adult IPD, albeit with some notable regional differences in the persistence of PCV13 serotypes and the prevalence of some emerging serotypes. In contrast to the relative homogeneity among these countries, a recent regional study from Japan did not find any case of serotype 8 or 9N IPD [26]. Another prominent difference is the USA, where no increases in non-PCV13 serotype IPD were evident in either children <5 years or adults ≥ 65 years and serotypes 8, 9N, 12F, and 15A were found to be responsible for a minority of IPD [27]. The reasons behind these differences continue to be a matter of debate [27].

Similarly to what was found in England [22], serotype 3 IPD in Portugal was associated with older age. This was also the case for serotypes 14, 22F, and 31 IPD. The increased importance of IPD by these serotypes with age could be associated with infections in adults with underlying conditions, whose proportion also increases with age [28], but our study was not designed to address this. The increased case fatality rate of serotype 3 IPD reported in England [22] and its increased prevalence in older-aged adults could also be partly responsible for the increasing mortality of pneumonia with age in Portugal [28].

Despite two decades of PCV7 use in children, PCV7 serotypes are still present as causes of IPD in adults, and their demise has not accelerated after four years of the introduction of PCV13 in the NIP with an uptake > 95%. Serotypes 14 and 19F (both PCV7 serotypes) are among the leading antimicrobial-resistant serotypes, suggesting that antimicrobial use could partly counter the selective force imposed by vaccination and justify their persistence. The proportion of IPD caused by PCV13 serotypes did decrease after introduction in the NIP but seems to have stabilized in the two most recent years, as in other European countries [20]. The future PCV15 vaccine covers and additional 8% of adult IPD relative to PCV13, and PCV20 covers an additional 28% relative to PCV15 (only 8% less than PPV23). The changes in the potential coverage of PCV20 and PPV23 varied in parallel, attesting to the relevance of the addPCV20 serotypes, which increased in importance in the ≥65 years group during the study period. The large proportion of vaccine-preventable disease in Portugal highlights the potential benefits of increasing the uptake of pneumococcal vaccines in adults, with the forthcoming PCV15 and, particularly, PCV20 vaccines broadening the potential coverage by PCVs and being welcome additions to the prevention of pneumococcal disease.

The average number of isolates from adult IPD sent to us per year in this study period (*n* = 543) substantially increased when compared to 2008–2014 (*n* = 405) [1]. Though this could have been due to increases in adult IPD, as reported elsewhere [21], it could also have been influenced by increased reporting. Consistent with the latter idea, there were no differences in the proportion of isolates of each age group between the two periods. Since our study was not population-based, we could not further clarify this point.

Our study had several limitations. Our surveillance was not population-based, and it was not designed to estimate the incidence of IPD because it was an exclusively laboratory surveillance and lacked compliance audits. A survey of hospitalized pneumonia cases among adults in Portugal in 2015 found that 1328 had pneumococcal etiology [28]. Considering that most cases of IPD are from invasive pneumonia (e.g., 70% of cases in the recent report from England [22]) and that invasive pneumonia is ≈10% of all pneumonia cases, the 530 isolates we received in 2015 represent a very high fraction of all adult IPD cases in Portugal. The stability of our surveillance network and its active nature are consistent with the identification of most IPD cases, as suggested by the available data for 2015, so we expect our sample to be representative of Portugal. The study was also not designed to collect information that is important to evaluate the severity of the infections caused by the different serotypes (e.g., hospitalization, ICU admission, and 30-day mortality) or relevant patient information (e.g., vaccination and comorbidities), which would have been important to better understand the changes in IPD accompanying the described serotype dynamics and the potential benefits of the currently used vaccines.

## 5. Conclusions

Despite the introduction of PCV13 in the NIP for children four years ago, the reduction in the PVC13 serotype adult IPD was modest and seems to have stabilized. At best, the herd effect may cause an ongoing slow decrease of addPCV13 serotype IPD like what we saw for the PCV7 serotypes, with almost two decades of use of PCVs targeting these serotypes with an uptake > 60%. This would mean that a substantial fraction of potentially vaccine-preventable disease would continue to occur in the coming years. The advent of new PCVs with a higher valency (PCV15 and PCV20), together with PPV23, affords new opportunities to prevent IPD in adults. An increase in vaccine uptake in adults could potentially lead to important reductions in the 12% mortality estimated for pneumococcal pneumonia requiring hospitalization in Portugal in 2015 [28].

## Figures and Tables

**Table 1:** Serotype distribution in each age group (*n* > 20 isolates).

Serotype	No of Isolates (%)			CA ^1^
	18–49 Years	50–64 Years	≥65 Years	
8	120 (30.2)	116 (23.3)	177 (13.8)	**<0.001**
3	40 (10.1)	82 (16.5)	212 (16.6)	**0.005**
22F	18 (4.5)	17 (3.4)	113 (8.8)	**<0.001**
14	15 (3.8)	28 (5.6)	95 (7.4)	**0.007**
19A	17 (4.3)	20 (4.0)	79 (6.2)	0.068
9N	25 (6.3)	19 (3.8)	52 (4.1)	0.109
11A	10 (2.5)	23 (4.6)	44 (3.4)	0.682
20	17 (4.3)	13 (2.6)	41 (3.2)	0.455
15A	5 (1.3)	12 (2.4)	39 (3.0)	0.050
19F	7 (1.8)	13 (2.6)	36 (2.8)	0.276
6C	3 (0.8)	7 (1.4)	42 (3.3)	**0.001**
7F	13 (3.3)	6 (1.2)	20 (1.6)	0.067
15B/C	8 (2.0)	8 (1.6)	23 (1.8)	0.863
23A	7 (1.8)	9 (1.8)	23 (1.8)	0.972
10A	7 (1.8)	10 (2.0)	21 (1.6)	0.765
16F	7 (1.8)	8 (1.6)	20 (1.6)	0.792
29/35B	3 (0.8)	5 (1.0)	25 (2.0)	0.053
31	1 (0.3)	5 (1.0)	26 (2.0)	**0.006**
12F	4 (1.0)	11 (2.2)	17 (1.3)	0.998
35F	9 (2.3)	6 (1.2)	15 (1.2)	0.145
4	10 (2.5)	10 (2.0)	8 (0.6)	**0.001**
23F	6 (1.5)	7 (1.4)	15 (1.2)	0.564
24F	3 (0.8)	4 (0.8)	17 (1.3)	0.262
33F	2 (0.5)	6 (1.2)	15 (1.2)	0.324

^1^ CA: Cochran–Armitage test for trend. In bold are the serotypes with significant *p*-values (*p* < 0.05) after FDR correction.

**Table 2:** Serotypes of the isolates responsible for invasive pneumococcal disease in adult patients (≥18 years) from 2012 to 2018.

Serotype ^1^	No. of Isolates (%)							CA ^2^	CA ^2^
				Current Study Period					
	2012	2013	2014	2015	2016	2017	2018	2015–2018	2012–2018
PCV13									
1	12 (3.0)	7 (1.8)	7 (1.9)	2 (0.4)	2 (0.4)	0 (0.0)	0 (0.0)	0.063	**<0.001**
3	66 (16.3)	45 (11.7)	50 (13.3)	72 (13.6)	79 (15.8)	84 (14.6)	99 (17.5)	0.125	0.154
4	6 (1.5)	8 (2.1)	9 (2.4)	7 (1.3)	8 (1.6)	8 (1.4)	5 (0.9)	0.478	0.171
6A	2 (0.5)	1 (0.3)	4 (1.1)	4 (0.8)	3 (0.6)	1 (0.2)	4 (0.7)	0.698	0.937
6B	5 (1.2)	5 (1.3)	5 (1.3)	3 (0.6)	1 (0.2)	6 (1.0)	1 (0.2)	0.795	0.031
7F	33 (8.2)	18 (4.7)	10 (2.7)	16 (3.0)	9 (1.8)	7 (1.2)	7 (1.2)	0.020	**<0.001**
9V	4 (1.0)	4 (1.0)	1 (0.3)	7 (1.3)	1 (0.2)	3 (0.5)	4 (0.7)	0.340	0.344
14	29 (7.2)	26 (6.8)	18 (4.8)	39 (7.4)	41 (8.2)	31 (5.4)	27 (4.8)	0.024	0.196
18C	1 (0.2)	4 (1.0)	2 (0.5)	3 (0.6)	3 (0.6)	5 (0.9)	2 (0.4)	0.795	0.998
19A	39 (9.7)	24 (6.3)	21 (5.6)	36 (6.8)	27 (5.4)	37 (6.4)	16 (2.8)	0.011	**<0.001**
19F	9 (2.2)	12 (3.1)	6 (1.6)	17 (3.2)	12 (2.4)	12 (2.1)	15 (2.7)	0.518	0.964
23F	1 (0.2)	3 (0.8)	9 (2.4)	6 (1.1)	8 (1.6)	4 (0.7)	10 (1.8)	0.632	0.213
addPPV23									
8	34 (8.4)	43 (11.2)	46 (12.2)	79 (14.9)	92 (18.4)	117 (20.3)	125 (22.1)	0.002	**<0.001**
9N	8 (2.0)	13 (3.4)	18 (4.8)	24 (4.5)	19 (3.8)	23 (4.0)	30 (5.3)	0.455	0.039
10A	2 (0.5)	8 (2.1)	8 (2.1)	9 (1.7)	11 (2.2)	8 (1.4)	10 (1.8)	0.815	0.486
11A	16 (4.0)	18 (4.7)	15 (4.0)	19 (3.6)	17 (3.4)	14 (2.4)	27 (4.8)	0.453	0.626
12F	6 (1.5)	8 (2.1)	4 (1.1)	5 (0.9)	3 (0.6)	9 (1.6)	15 (2.7)	0.008	0.349
15B/C	5 (1.2)	9 (2.3)	8 (2.1)	7 (1.3)	8 (1.6)	16 (2.8)	8 (1.4)	0.578	0.756
17F	5 (1.2)	2 (0.5)	2 (0.5)	5 (0.9)	2 (0.4)	3 (0.5)	8 (1.4)	0.371	0.775
20	14 (3.5)	11 (2.9)	14 (3.7)	18 (3.4)	17 (3.4)	23 (4.0)	13 (2.3)	0.420	0.665
22F	25 (6.2)	23 (6.0)	31 (8.2)	40 (7.5)	34 (6.8)	45 (7.8)	29 (5.1)	0.191	0.759
33F	1 (0.2)	1 (0.3)	1 (0.3)	1 (0.2)	6 (1.2)	7 (1.2)	9 (1.6)	0.031	**0.001**
NVT									
6C	8 (2.0)	14 (3.7)	6 (1.6)	20 (3.8)	14 (2.8)	8 (1.4)	10 (1.8)	0.011	0.212
7C	1 (0.2)	4 (1.0)	1 (0.3)	2 (0.4)	2 (0.4)	5 (0.9)	5 (0.9)	0.196	0.308
15A	3 (0.7)	11 (2.9)	13 (3.5)	12 (2.3)	14 (2.8)	14 (2.4)	16 (2.8)	0.660	0.235
16F	13 (3.2)	3 (0.8)	7 (1.9)	11 (2.1)	8 (1.6)	13 (2.3)	3 (0.5)	0.095	0.079
23A	9 (2.2)	8 (2.1)	9 (2.4)	13 (2.5)	7 (1.4)	12 (2.1)	7 (1.2)	0.161	0.159
23B	4 (1.0)	5 (1.3)	3 (0.8)	6 (1.1)	3 (0.6)	4 (0.7)	6 (1.1)	0.951	0.632
24F	5 (1.2)	9 (2.3)	9 (2.4)	7 (1.3)	7 (1.4)	5 (0.9)	5 (0.9)	0.359	0.075
25A/38	3 (0.7)	3 (0.8)	2 (0.5)	1 (0.2)	3 (0.6)	5 (0.9)	1 (0.2)	0.870	0.448
29/35B	10 (2.5)	6 (1.6)	10 (2.7)	6 (1.1)	7 (1.4)	14 (2.4)	10 (1.8)	0.241	0.709
31	5 (1.2)	2 (0.5)	4 (1.1)	6 (1.1)	10 (2.0)	9 (1.6)	7 (1.2)	0.956	0.309
33A	2 (0.5)	5 (1.3)	2 (0.5)	4 (0.8)	0 (0.0)	0 (0.0)	1 (0.2)	0.060	0.010
34	3 (0.7)	1 (0.3)	4 (1.1)	5 (0.9)	3 (0.6)	1 (0.2)	3 (0.5)	0.245	0.431
35F	7 (1.7)	4 (1.0)	2 (0.5)	5 (0.9)	11 (2.2)	6 (1.0)	8 (1.4)	0.904	0.808
NT	1 (0.2)	3 (0.8)	6 (1.6)	2 (0.4)	1 (0.2)	3 (0.5)	2 (0.4)	0.834	0.380
Other	7 (1.7)	12 (3.1)	9 (2.4)	11 (2.1)	8 (1.6)	14 (2.4)	18 (3.2)		
Total	404	383	376	530	501	576	566	-	-

^1^ Only serotypes detected in >3 isolates in at least one year are shown; the remaining are represented in “others.” PCV13: serotypes included in PCV13; addPPV13: the additional serotypes included in PPV23 that are not present in PCV13; NVT: non-vaccine types. ^2^ CA: Cochran–Armitage test for trend. In bold are the serotypes with significant *p*-values (*p* < 0.05) after FDR correction.

**Table 3:** Number of isolates responsible for invasive pneumococcal disease in adult patients, according to vaccine serotype groups and age groups, from 2015 to 2018.

Age Group (Years)	Serotype Group ^1^	No. of Isolates (%)				CA ^2^
		2015	2016	2017	2018	
18–49	PCV7	8 (7.8)	14 (16.5)	12 (10.9)	8 (8.1)	0.807
	addPCV13	23 (22.3)	19 (22.4)	15 (13.6)	22 (22.2)	0.593
	addPCV15	13 (12.6)	3 (3.5)	4 (3.6)	0 (0)	**<0.001**
	addPCV20	35 (34.0)	28 (32.9)	44 (40.0)	42 (42.4)	0.139
	addPPV23	11 (10.7)	8 (9.4)	13 (11.8)	15 (15.2)	0.286
	NVT	13 (12.6)	13 (15.3)	22 (20.0)	12 (12.1)	0.802
50–64	PCV7	21 (16.9)	16 (14.0)	18 (14.4)	15 (11.2)	0.216
	addPCV13	33 (26.6)	26 (22.8)	24 (19.2)	27 (20.1)	0.166
	addPCV15	2 (1.6)	7 (6.1)	7 (5.6)	7 (5.2)	0.218
	addPCV20	30 (24.2)	44 (38.6)	44 (35.2)	51 (38.1)	0.041
	addPPV23	13 (10.5)	3 (2.6)	6 (4.8)	13 (9.7)	0.993
	NVT	25 (20.2)	18 (15.8)	26 (20.8)	21 (15.7)	0.558
≥65	PCV7	53 (17.5)	44 (14.6)	39 (11.4)	41 (12.3)	0.033
	addPCV13	74 (24.4)	75 (24.8)	90 (26.4)	77 (23.1)	0.819
	addPCV15	26 (8.6)	30 (9.9)	41 (12.0)	31 (9.3)	0.584
	addPCV20	54 (17.8)	59 (19.5)	78 (22.9)	92 (27.6)	**0.002**
	addPPV23	23 (7.6)	27 (8.9)	30 (8.8)	23 (6.9)	0.733
	NVT	73 (24.1)	67 (22.2)	63 (18.5)	69 (20.7)	0.183

^1^ PCV7: serotypes included in the 7-valent conjugate vaccine; addPCV13: additional serotypes included in the 13-valent conjugate vaccine and not present in PCV7—1, 3, 5, 6A, 7F, and 19A; addPCV15: additional serotypes included in the 15-valent conjugate vaccine and not present in PCV13—22F and 33F; addPCV20: additional serotypes included in the 20-valent conjugate vaccine and not present in PCV15—8, 10A, 11A, 12F, and 15B (for the purpose of this paper, we assumed that protection was afforded against IPD by the 15B/C group of isolates); addPPV23: the additional 3 serotypes exclusively present in the 23-valent pneumococcal polysaccharide vaccine—9N, 17F and 20; NVT: serotypes not included in any of the currently available pneumococcal vaccines. ^2^ CA: Cochran–Armitage test for trend. Values in bold were significant after FDR correction.

**Table 4:** Number of isolates responsible for invasive pneumococcal disease in adult patients, according to vaccine serotype groups and age groups, from 2015 to 2018.

	No. Resistant Isolates (%)		
Antimicrobial ^1^	18–49 Years (*n* = 397)	50–64 Years (*n* = 497)	≥65 Years (*n* = 1278) ^2^
PEN	44 (11.1)	65 (13.8)	217 (17.0)
MIC90 ^3^	0.016	0.125	0.5
MIC50 ^3^	0.012	0.012	0.012
CTX	1 (0.3)	2 (0.4)	3 (0.2)
MIC90 ^3^	0.016	0.125	0.38
MIC50 ^3^	0.016	0.016	0.016
LEV	2 (0.5)	2 (0.4)	9 (0.7)
ERY	51 (12.8)	57 (11.5)	214 (16.7)
CLI	41 (10.3)	49 (9.9)	185 (14.5)
CHL	5 (1.3)	12 (2.4)	23 (1.8)
SXT	47 (11.8)	62 (12.5)	175 (13.7)
TET	53 (13.4)	79 (15.9)	214 (16.7)

^1^ PEN: penicillin; CTX: cefotaxime; LEV: levofloxacin; ERY: erythromycin; CLI: clindamycin; CHL: chloramphenicol; SXT: trimethoprim/sulfamethoxazole; TET: tetracycline. All isolates were susceptible to vancomycin and linezolid. ^2^ In this age group, it was not possible to determine the antimicrobial resistance in the case where the diagnosis was made by molecular methods. ^3^ The minimal inhibitory concentration values of 50% or 90% of the isolates are shown in mg/L.

## Data Availability

All the data in this study is available in the figures and tables of the paper.
